# Whole-Exome Analysis and Osteosarcoma: A Game Still Open

**DOI:** 10.3390/ijms252413657

**Published:** 2024-12-20

**Authors:** Caterina Chiappetta, Carlo Della Rocca, Claudio Di Cristofano

**Affiliations:** 1 AOU Policlinico Umberto I, 00161 Rome, Italy; caterina.chiappetta@uniroma1.it; 2 Department of Medico-Surgical Sciences and Biotechnologies, Sapienza University of Rome, 04100 Latina, Italy; carlo.dellarocca@uniroma1.it

**Keywords:** osteosarcoma, next-generation sequencing, tumor targeted therapy, immunotherapy

## Abstract

Osteosarcoma (OS) is the most prevalent malignant bone tumor in adolescents and young adults. OS cells grow in a permissive local microenvironment which modulates their behavior and facilitates all steps in tumor development (e.g., proliferation/quiescence, invasion/migration, and drug resistance) and contributes to their intrinsic heterogeneity. The lung parenchyma is the most common metastatic site in OS, and metastatic foci are frequently associated with a poor clinical outcome. Although multiple factors may be responsible for the disease, including genetic mutations (e.g., *Rb* and *p53*), the molecular mechanism of development of OS remains unclear, and the conventional treatment for OS is still based on a sequential approach that combines chemotherapy and surgery. Also, despite the increase in clinical trials, the survival rates for OS have not improved. Non-specific targeting therapies thus show poor therapeutic effects, along with side effects at high doses. For these reasons, many efforts have been made to characterize the complex genome of OS thanks to the whole-exome analysis, with the aim of identifying predictive biomarkers to give these patients a better therapeutic option. This review aims to summarize and discuss the main recent advances in OS molecular research for precision medicine.

## 1. Introduction

OS is the most common nonhematological primary malignant tumor of the bone. It arises from mesenchymal cells that produce osteoid and immature bone and affects mainly the extremities of adolescents and young adults [1,2]. Its biologic behavior involves various factors, such as aggressiveness: OS is highly aggressive and tends to grow rapidly. It can metastasize to other organs, commonly the lungs. Local invasion: It has the capacity to invade nearby tissues and structures, causing bone destruction and potential fractures. High recurrence risk: Even after treatment, there is a risk of recurrence in OS patients, particularly if the tumor was not completely removed or if cells have spread to other areas of the body [3]. OS is a type of bone tumor that has a complex and multifactorial pathogenesis [4,5]. Although it is not fully understood, there are some key factors that contribute to its formation:

- Pre-existing bone lesions: OS can develop following pre-existing lesions to the bones, such as trauma or bone pathologies.
- Hereditary factors: In some cases, OS may be associated with rare hereditary conditions that increase the risk of developing this type of tumor, such as hereditary retinoblastoma. Indeed, mutations in the *RB1* gene have been commonly associated with hereditary OS. The RB1 protein plays a crucial role in cell cycle control, and its dysfunction can lead to uncontrolled cell proliferation, a common trait in tumors [6]. For example, Li–Fraumeni syndrome is linked to mutations in the *TP53* gene, which is involved in tumor suppression. People with this syndrome have a higher risk of developing several types of tumors, including OS [7]. Other genetic conditions, such as Rothmund–Thomson syndrome, Bloom syndrome, and Werner syndrome, can increase the risk of developing OS [8].
- Radiation exposure: Ionizing exposure to high doses of radiation may increase the risk of developing OS.
- Rapid growth and development: Because OS often affects growing young people, it is thought that rapid bone growth and development may play a role in its formation.
- Genetic mutations: Genetic alterations, such as mutations in the *TP53* and *RB1* genes and Wnt signaling pathways, are associated with the development of OS. These mutations can alter the control of cell growth, favoring the formation of tumor cells [4,5].

The treatment of high-grade OS is based on a multidisciplinary approach that includes neoadjuvant chemotherapy, surgical excision of the primary tumor, and metastasis excision; evaluation of response to therapy in the surgical specimen is crucial to eventually schedule a postoperative chemotherapy [9]. Patients’ survival is related to the development of metastasis and the response to chemotherapy. Standard therapy regimens often involve the use of high-dose methotrexate, doxorubicin, cisplatin, and ifosfamide [10,11]. Moreover, OS cases are commonly resistant to traditional chemotherapies, and high-dose chemotherapy results in severe side effects [12].

Some of the predictive markers and prognostic factors that are considered include the following:

- Tumor grade: Classifying the tumor based on the degree of aggressiveness can provide information on the growth rate and potential of the cancer to spread.
- Extension of the tumor: The size of the tumor and whether it has spread to surrounding tissues can influence treatment and prognosis.
- Metastasis: The presence or absence of metastases, particularly in the lungs, is an important prognostic factor for OS. Indeed, in patients with metastatic OS treated with neoadjuvant therapy, the “responder” status shows improved survival (82% at 5-years) compared to “non-responder” (70% at 5-years) [13,14].
- Response to neoadjuvant chemotherapy: The response of the tumor to chemotherapy administered before surgery can be a prognostic indicator. A good response may indicate a better prognosis.
- Age: The age of the patient at the time of diagnosis can influence the prognosis. For example, younger patients tend to respond better to treatment.
- Tumor location: The specific location of the tumor within the bone may have prognostic implications [15].

Moreover, some genetic mutations can influence tumor behavior and response to treatment. For example, the presence of mutations in the *TP53* or *RB1* genes may correlate with less favorable prognoses [4,5]. However, the somatic genome of the OS is considered complex and characterized by tumor heterogeneity [16,17]; indeed, an increased number of mutations, not only in *TP53* or *RB1* genes, but also in genes that are part of the Wnt signaling pathway, such as APC (adenomatous polyposis coli) and β-catenin, have been associated with OS. This signaling pathway regulates cell growth and differentiation [18]. Mutations in genes involved in the MAP kinase signaling pathway, such as *BRAF*, may be present in some subtypes of OS, affecting the growth and survival of tumor cells [19] and the mutation rate of OS is the highest among all pediatric tumors [16,17]. In addition to point mutations, OS can present structural genomic changes, such as copy number variations, chromosomal rearrangements, deletions, or amplifications, that can affect the function of key genes in the control of cell growth [20]. Also, the gene expression analysis can identify genes that are overexpressed or downregulated in tumor cells [5], and alterations in cellular signaling pathways, such as the insulin-like growth factor (*IGF*) pathway or the epidermal growth factor (EGF) pathway, may be involved in the growth and survival of tumor cells [21]. Furthermore, phenomena of chromothripsis (massive genomic rearrangement that occurs in a single event) and kataegis (localized hypermutation) have been demonstrated in OSs. Kataegis was found in over 50% of OSs analyzed with whole-genome sequencing [22]. Nonetheless, survival rates of patients have not greatly improved [20], because these alterations can vary from patient to patient, contributing to the diversity of the disease and making it difficult to identify a single cause or pathogenetic pathway. Moreover, a recent study [23] suggests that the development of cancer stem cells (CSCs) could be the reason for chemotherapeutic resistance, and it has been reported that these CSCs are regulated by noncoding RNAs (ncRNAs) especially long non-coding RNAs (lncRNAs) [24]. CSCs play a key role in tumor development, progression, and drug resistance, so the identification of ncRNAs could be important to identify CSCs in OS and so considering these ncRNAs as potential biomarkers.

Therefore, identification of alterations in coding and noncoding genome of OS is essential to better understand tumor biology and develop targeted therapies that can stop specific molecular pathways involved in its growth and spread, resulting in a lack of more effective and tailored chemotherapy drug regimens [25].

So, identifying potential prognostic and predictive markers in OS involves exploring various avenues of research and analysis, and different areas of investigation are explored [26]:

- Clinical data analysis involves analyzing large datasets of clinical information from OS patients to identify patterns or correlations between demographic factors, treatment protocols, and patient outcomes. This could involve retrospective studies or meta-analyses of existing clinical data [27].
- Radiological and imaging markers involve using advanced imaging techniques like MRI, PET-CT scans, or other imaging modalities to identify specific radiological markers associated with tumor aggressiveness, response to treatment, or recurrence. Changes in tumor characteristics visible on imaging might provide insights into prognosis [28].
- Immunohistochemistry studies involve examining tissue samples from OS patients to identify specific protein markers or antigen expressions associated with disease behavior or response to treatment. Immunohistochemistry studies can reveal valuable information about the tumor immune microenvironment, and it has become a recent research hot spot, providing valuable insight into tumor heterogeneity that could influence disease progression [29]. Indeed, in a recent study based on single-cell RNA sequencing (scRNA-seq), He and collaborators showed how changes in the cytotoxicity and immune checkpoint gene expression of CD8+ T cells in OS lung metastasis could explain the complexity of tumor microenvironment of OS lung metastasis, making it possible precision therapeutic approaches [30].
- Molecular biomarkers involve investigating specific genetic mutations or molecular markers associated with OS progression, response to treatment, or recurrence. This also involves analyzing gene expression profiles, identifying oncogenes or tumor suppressor genes, or exploring epigenetic modifications [26,31]. Also, searching for circulating biomarkers in blood, urine, or other bodily fluids that can indicate disease progression, treatment response, or recurrence is involved. This involves analyzing proteins, circulating tumor cells, and circulating tumor DNA (ctDNA) or microRNAs [32].
- Drug sensitivity and resistance studies involve investigating factors that contribute to drug resistance or sensitivity in OS treatments. Understanding why certain tumors respond differently to therapies can lead to the identification of predictive markers [33].
- Multi-Omics approaches involve integrating data from genomics, proteomics, metabolomics, and other omics fields to comprehensively understand the complex molecular landscape of OS. This holistic approach might unveil novel markers or pathways relevant to prognosis and treatment response [34].
- Machine learning and artificial intelligence involve employing computational methods to analyze complex datasets and identify potential prognostic or predictive markers. Machine learning algorithms can help in discovering patterns and associations that might not be immediately apparent through traditional analysis methods [35].

So, we carried out a very thorough review of the literature regarding advances in understanding the pathogenesis of OS. Above all, we focused on molecular studies aimed at finding biomarkers that could have therapeutic implications. In addition, we researched the latest clinical trials concerning this pathology. All this information allowed us to have a snapshot of the State of the Art of the recent advances in this field.

## 2. Relevant Sections

Precision medicine may involve analyzing the tumor’s genetic profile to identify specific mutations or molecular alterations present in tumor cells. This information can be used to identify specific therapeutic targets within tumor cells, enabling the targeted use of drugs or therapies that may be most effective against those specific mutations or markers to allow the choice of treatments best suited to the molecular profile of a patient’s tumor, avoiding treatments that may not be effective or potentially harmful, and using molecular testing to monitor the tumor’s response to treatment over time and make any therapeutic changes or adjustments based on the patient’s response. The increasing use of Next-Generation Sequencing (NGS) has completely revolutionized clinical research over the last decade [36,37]. In this context, Whole-Exome Sequencing (WES) is a high-throughput sequencing technique that focuses on sequencing the exonic regions of the genome—those parts of DNA that encode proteins. The exome represents only about 1–2% of the entire genome, but it contains approximately 85% of known disease-related variants [38]. WES is widely used in both clinical and research settings to identify genetic variations that may be associated with various diseases, including cancers [39]. In WES, DNA is first extracted from the cells of interest, followed by the selective capture of exonic regions using hybridization techniques, such as probes complementary to known exonic sequences. Once the exonic DNA is isolated, high-throughput sequencing technologies generate millions of short DNA reads, which are then aligned to a reference genome. After sequencing, the data are analyzed to identify mutations, such as single-nucleotide polymorphisms (SNPs), insertions, deletions, and other genomic alterations that might be significant for disease [40]. WES has become a powerful tool in cancer research, providing insights into the genetic underpinnings of cancer, identifying potential therapeutic targets, and aiding in the development of personalized medicine strategies. Particularly, WES enables the identification of mutations in oncogenes and tumor suppressor genes, which are often found in cancerous cells [41]. Moreover, WES allows researchers to study the diversity of mutations within a tumor, which is important for understanding cancer progression and developing effective treatments. Additionally, comparing the somatic mutations in tumors versus normal tissues can help identify actionable mutations that drive cancer. WES can help identify mutations that may be targeted by specific therapies, and it helps create comprehensive genomic profiles of tumors, identifying both common and rare mutations. This data can guide clinicians in selecting the most appropriate treatment options based on the genetic makeup of a patient’s cancer, facilitating personalized cancer therapy and improving outcomes [42]. WES can also be used to study the tumor microenvironment, where interactions between cancer cells, immune cells, and stromal cells play a crucial role in cancer progression and metastasis. By analyzing the mutations present in both tumor and immune cells, researchers can understand how tumors evade immune detection and response [43]. Also, WES can help identify genetic markers that are associated with early-stage cancers or high-risk tumors. By detecting mutations or genetic signatures that precede cancer development, WES can potentially be used for early detection. Additionally, certain mutations may be prognostic of disease outcome, providing valuable information about a patient’s prognosis. Finally, studying the mutations found in recurrent or metastatic tumors using WES can provide insights into how cancer evolves during treatment. This can help identify mutations that contribute to drug resistance, allowing for the development of second-line therapies or combination treatments to overcome resistance [44].

Particularly in OS, WES can be used to better understand the genetic causes underlying the development of this disease [45] thanks to the integration of molecular and clinical data to obtain a more complete and accurate view of the disease and develop personalized therapeutic approaches.

There are many examples of “molecular targeted therapy”, where tailored therapeutic agents have been selected to aim against specific molecules and their downstream effector pathways in each patient [46]. Tyrosine kinase inhibitors (TKIs) have been explored in the context of OS treatment due to their ability to target specific pathways involved in cancer growth and progression [47]. TKIs are a class of drugs that work by blocking the activity of specific enzymes called tyrosine kinases. These enzymes are involved in various cellular processes, including cell growth and proliferation. By inhibiting these kinases, TKIs can potentially impede the growth and spread of cancer cells. Several TKIs have been studied in the context of OS, either as standalone treatments or in combination with other therapies. Drugs like sorafenib, sunitinib, and dasatinib are examples of TKIs that have shown some promise in preclinical studies or early-phase clinical trials for OS [47]. However, while there have been some encouraging results in laboratory studies and early trials, the effectiveness of TKIs in treating OS in larger clinical settings is still under investigation. Challenges remain, including issues related to drug resistance, side effects, and the need for more extensive clinical data to establish their efficacy and safety [48]. As research advances and more clinical data become available, the role of TKIs in OS treatment may become clearer, potentially offering new avenues for improved therapies and outcomes for patients.

Anti-angiogenesis agents are drugs that inhibit the formation of new blood vessels. In the context of cancer, these drugs aim to prevent the growth of new blood vessels that tumors need to thrive and spread [49]. OS is known for its highly vascularized nature, meaning it has a significant network of blood vessels supplying it with nutrients and oxygen [50]. Anti-angiogenesis therapy might be a promising approach in treating OS because these agents can potentially hinder its growth and metastasis, but the efficacy of anti-angiogenesis agents alone in treating OS might be limited. They are often used in combination with other treatments, like chemotherapy or targeted therapies, for more effective results. Drugs like bevacizumab and sorafenib are among those that have been studied for their anti-angiogenic properties in OS. Clinical trials are ongoing to evaluate their effectiveness, both alone and in combination with other therapies, in improving outcomes for patients with OS. While there is promise in utilizing anti-angiogenesis agents, further research is needed to determine their optimal use, potential side effects, and long-term benefits in treating OS [51].

The mTOR pathway plays a significant role in the OS disease. mTOR (mammalian target of rapamycin) is a protein that regulates various cellular processes, including cell growth, proliferation, and survival. In OS, there is often dysregulation or overactivity in the mTOR pathway, contributing to the uncontrolled growth of cancer cells [52]. The abnormal activation of mTOR signaling can lead to increased cell proliferation, resistance to cell death, and the promotion of tumor growth. As a result, targeting the mTOR pathway has been a subject of interest in the development of potential treatments for OS [53]. mTOR inhibitors, such as everolimus and sirolimus, aim to block or reduce the activity of mTOR, thereby potentially slowing down the growth and spread of OS cells [52]. However, the effectiveness of mTOR inhibitors in treating OS is an active area of research and often involves combination therapies or clinical trials to assess their efficacy [54].

Moreover, immunotherapy has been an area of interest in the treatment of OS [55]. Checkpoint Inhibitors help the immune system recognize and attack cancer cells by targeting proteins that inhibit immune responses; drugs like pembrolizumab and nivolumab have been investigated in clinical trials for OS [56]. Vaccine therapies aim to stimulate the body’s immune system to recognize and attack cancer cells, and some studies have explored vaccine-based approaches for OS [57]. Chimeric Antigen Receptor (CAR) T-Cell Therapy is a type of adoptive cell therapy where a patient’s own immune cells are modified to better recognize and attack cancer cells; while this has seen success in some blood cancers, its efficacy in solid tumors like OS is an area of ongoing research [58]. Immunotherapy for OS is still in its early stages compared to its use in other cancers, and while there have been some promising results, more research is needed to understand its effectiveness, potential side effects, and the best ways to combine it with existing treatments. Clinical trials continue to explore these avenues to improve outcomes for individuals with OS [57]. Indeed, there are several ongoing clinical trials exploring new therapies and treatment strategies for OS. These trials aim to evaluate the safety and efficacy of novel drugs, combination therapies, and innovative treatment approaches. EURAMOS-1 (NCT00134030), although initiated over a decade ago, is a landmark international study that continues to generate valuable data. This trial evaluates the addition of the bisphosphonate zoledronic acid to standard chemotherapy in patients with localized OS. The goal is to assess whether zoledronic acid can improve event-free survival in this population [59]. The INFORM registry trial (NCT03838042) aims to collect comprehensive molecular and clinical data from pediatric and adolescent patients with rare tumors, including OS. By analyzing tumor samples for genetic alterations and other molecular characteristics, researchers seek to identify potential targets for precision therapies and improve understanding of the underlying biology of OS [60]. SARC024 (NCT02048371), a phase II trial, evaluates the efficacy of the VEGFR inhibitor regorafenib in patients with recurrent or refractory OS. The study aims to assess the objective response rate and progression-free survival associated with regorafenib treatment in this patient population [61]. Various phase II trials conducted by the Children’s Oncology Group (COG Phase II Trials) are exploring novel agents and treatment approaches for OS. Examples include a trial investigating the efficacy of the immunotherapy agent nivolumab (NCT02304458) in patients with relapsed or refractory OS [62]. These trials represent a snapshot of the ongoing efforts to improve outcomes for patients with OS (Table 1).

Moreover, epigenetic alterations play a crucial role in OS progression. Unlike genetic mutations, epigenetic changes can modify gene expression without altering the underlying DNA sequence [63]. Histone deacetylases (HDACs) are key enzymes in this process, responsible for removing acetyl groups from histone proteins and typically associated with gene silencing and chromatin condensation. Particularly, HDAC inhibitors work by preventing the removal of acetyl groups from histone proteins, promoting a more open chromatin structure, reactivating potentially silenced tumor suppressor genes, and disrupting cancer cell proliferation and survival mechanisms. In this way, HDAC inhibitors can trigger cell cycle arrest, preventing uncontrolled cancer cell proliferation; they can promote programmed cell death in OS cells; and they can potentially enhance the effectiveness of existing chemotherapy treatments by making cancer cells more vulnerable [64,65]. So, targeting the cancer epigenome with HDAC inhibitors represents a cutting-edge approach in OS treatment. While significant challenges remain, the potential for developing more personalized and effective therapeutic strategies is promising, and researchers aim to advance the field of OS treatment and ultimately improve survival rates and quality of life for OS patients.

## 3. Discussion

Besides the advance in the field of predictive biomarkers for cancer therapy in the last decades, OS is a so-called “orphan cancer” with no known driver oncogenes [66]. Some studies based on the NGS approach were performed to better understand the complex biology of this tumor and the molecular pathways that lead to the development of metastases and resistance to therapy [67]. Surely, studies on exome sequencing in OS have significantly contributed to our understanding of the genetic landscape and molecular mechanisms underlying this bone cancer; nevertheless, exome sequencing studies have revealed a complex mutational landscape in OS, characterized by a wide range of genetic alterations [26]. In our previous studies [45,68], we performed a WES analysis on high OS biopsies obtained before neoadjuvant therapy, confirming the complexity of OS karyotype [26]. We found that the *KMT2C* gene, a key component of histone H3 lysine 4 methyltransferase complexes [69], showed the highest number of variations in most of the samples being analyzed. *KMT2C* is mutated in a wide spectrum of neoplasms; it has been linked to tumorigenesis [69]; and some studies suggest that variations in coding sequences of regulating elements, which act on enhancers to recognize specific transcriptional factors, may be the cause of tumor development [70]. Indeed, these modifications can activate or repress gene expression, influencing cell behavior. Alterations in *KMT2C* can disrupt this regulation, leading to abnormal expression of genes involved in cell growth, division, and differentiation, which can contribute to cancer development. Moreover, a recent study by Gaeta et al. [71] showed a prevalence of mutations in some genes, in particular, in genes involved in homologous recombination process by opening doors to the possibility to use the PARP inhibitors as a potential therapeutic option in OS patients; indeed, another previous study that analyzed all bone tumors showed a high frequency of HRD-related mutations through germline mutation analysis [72].

Not only HRD but also other biomarkers for immune checkpoint inhibitor response were studied in OS through WES. Indeed, a recent review [73] about sarcomas highlights how specific signatures, such as TMB (Tumor Mutational Burden), MSI (Microsatellite Instability) and deficiency, in *BRCA1/2* genes could be useful biomarkers in this type of tumor. Particularly, some studies observed that OSs that showed deficiency in *BRCA1/2* genes also presented specific genomic alterations, such as those seen in other PARP-sensitive tumors [74,75].

Regarding TMB, defined as total number of non-synonymous somatic mutations per megabase (muts/Mb) in coding areas harbored by tumor cells in a given neoplasm, another study [76] about the analysis of this biomarker through WES in OS supports the use of TMB to predict the prognosis in OS and so to be relevant in the treatment decision making process.

Also, the most common genetic alterations involved in cell cycle were frequently found in OS after WES analysis [72,76]. Despite the fact that some of the genes harboring genetic alterations are the same in adult and pediatric OSs, pediatric tumors presented more CNV (Copy Number Variation) and genetic fusion than SNVs (Single-Nucleotide Variations) and insertions and deletions. Indeed, a study focalized on the use of NGS for pediatric OS showed an association between specific genetic alterations and the patient’s age at time of diagnosis [77].

Moreover, a recent study showed how the WES analysis in OS is able to identify alteration in genes involved in the protein–protein networking on specific pathways implicated in skeletal system development [78].

So, it is clear that WES analysis plays a crucial role in understanding the genetic landscape of OS, and there are several reasons highlighting the importance of this approach in this type of cancer, such as the discovery of driver mutations for developing personalized treatment approaches, the identification of prognostic markers, and a better understanding of tumor heterogeneity. However, although WES is cheaper than WGS, it is still relatively expensive, particularly when used in large-scale clinical studies. Also, the large volume of data generated from WES requires sophisticated bioinformatics tools for accurate interpretation. False positives and false negatives can arise if the data are not carefully analyzed. Moreover, some exonic regions, especially those that are GC-rich or repetitive, can be poorly captured, leading to gaps in data that may miss critical mutations [79]. Future advancements in WES technology may address these challenges, with improvements in capture techniques, sequencing platforms, and bioinformatics pipelines. Additionally, integration with other omics approaches, such as transcriptomics and proteomics, will provide a more holistic understanding of cancer biology and enhance the clinical utility of WES. So, WES analysis in OS surely is instrumental for unraveling the complex genetic basis of the disease, guiding treatment decisions, and advancing our understanding of the underlying molecular mechanisms. This knowledge is essential for the development of more effective and personalized therapeutic approaches for patients with OS.

However, the significant molecular, genetic, and clinical heterogeneity of OS poses substantial obstacles to research and treatment development. This heterogeneity manifests across multiple dimensions, creating intricate challenges for researchers and clinicians. Indeed, each tumor can harbor unique genetic alterations, chromosomal abnormalities, and molecular signatures; this genetic variability means that two patients with seemingly similar clinical presentations may have fundamentally different underlying tumor characteristics [80]. Obviously, this inherent heterogeneity directly impacts research methodologies, creating several critical challenges. Traditional clinical trials and research studies struggle to effectively stratify patients due to the complex nature of OS. Researchers must develop more nuanced classification systems that go beyond basic clinical parameters and incorporate molecular and genetic insights. The significant variability between patient populations makes it difficult to establish statistically robust conclusions, generalize research findings, develop universally applicable treatment protocols, and create reliable predictive models. Also, researchers face substantial difficulties in designing representative study cohorts, controlling for multiple variables, ensuring meaningful comparisons between different patient groups, and developing standardized research protocols. So, the heterogeneous nature of OS directly impacts clinical outcomes and treatment strategies, and different molecular subtypes may respond differently to standard therapies (Table 2).

## 4. Conclusions and Future Directions

It is important to note that research in this field is constantly evolving; however, OS is a complex tumor, and treatment varies depending on the individual case. New therapies are emerging thanks to a better understanding of tumor biology and its molecular characteristics, paving the way for more targeted and effective treatment options. Participation in clinical trials can be an opportunity to access promising experimental treatments.

In our view, molecular studies that allow for extensive characterization of OS are fundamental for a deeper understanding of the pathogenesis of this disease. Translational medicine, especially for “orphan” tumors lacking targeted therapies like OS, represents a bridge between research and therapy for these patients. We believe that comprehensive molecular characterization performed at diagnosis on pre-treatment biopsies can provide insights with significant clinical and therapeutic implications. In fact, WES analysis has the potential to identify specific genetic variations in selected groups of patients who share common clinico-pathological features, leading to the generation of innovative prognostic/predictive molecular tools to be implemented in clinical practice. However, the advent of WES in oncology is generating great enthusiasm, but, currently, there is a partial lack of validation studies and comparisons between different platforms. The enormous amount of data generated requires complex bioinformatic analysis that also needs validation phases. Probably, the creation of a network of specialized centers for the diagnosis and treatment of OS will allow for the collection of a significant number of cases for retrospective and prospective studies in a field where rarity represents a major challenge for research and clinical practice. The availability of clinically and molecularly annotated histological samples is a crucial prerequisite for understanding the complex biological bases of OS genesis. The molecular annotation of OS can provide the rationale for the appropriate selection of patients to be treated with personalized approaches. In addition to better-directed therapy, appropriate selection implies a key impact on sustainability. Considering the rising cost of medical therapy in oncology, adequate patient selection seems mandatory to avoid the risk of patient discrimination due to a lack of resources.

Finally, clinical trials of OS treatment are crucial to develop new therapies and improve current treatment options. These studies involve volunteer patients and are conducted to evaluate the effectiveness and safety of new drugs, targeted therapies, surgical approaches, or combined treatment protocols. Also, successful identification of prognostic and predictive markers in OS often requires collaboration between clinicians, pathologists, geneticists, bioinformaticians, and researchers from various disciplines, because, to date, the heterogeneity of OS represents a significant scientific and clinical challenge. Overcoming these complexities requires interdisciplinary collaboration, advanced technological approaches, and a fundamental reimagining of research methodologies. This interdisciplinary approach allows for a more comprehensive exploration of the disease and increases the likelihood of discovering clinically relevant markers.

## Figures and Tables

**Table 1:** Ongoing clinical trials for patients with OS. The table includes data on the identification code, title, and who sponsored the study.

Clinical Trial	Clinical Trials ID	Sponsor	Official Title
EURAMOS-1	NCT00134030	Children’s Oncology Group	“A Randomized Trial of the European and American Osteosarcoma Study Group to Optimize Treatment Strategies for Resectable Osteosarcoma Based on Histological Response to Pre-Operative Chemotherapy”
INFORM2	NCT03838042	University Hospital Heidelberg	“INFORM2 Exploratory Multinational Phase I/II Combination Study of Nivolumab and Entinostat in Children and Adolescents with Refractory High-risk Malignancies (INFORM2-NivEnt)”
SARC024	NCT02048371	Sarcoma Alliance for Research through Collaboration	“SARC024: A Blanket Protocol to Study Oral Regorafenib in Patients with Selected Sarcoma Subtypes”
	NCT02304458	National Cancer Institute (NCI)	“A Phase 1/2 Study of Nivolumab in Children, Adolescents, and Young Adults with Recurrent or Refractory Solid Tumors as a Single Agent and in Combination with Ipilimumab”

**Table 2:** List of the important genes and complex biomarker features and their prevalence and clinical significance in OS [76,81,82,83,84,85,86,87].

Genes/Complex Biomarker	Prevalence	Clinical Significance
*TP53*	~50–60%	Tumor suppressor gene responsible for cell cycle regulation and apoptosis
		Mutations are associated with poor prognosis and increased tumor aggressiveness
		Loss of p53 function contributes to genomic instability and reduced DNA repair mechanisms
		Often an early event in OS tumorigenesis
*RB1*	~30–40%	Regulates cell cycle progression and prevents uncontrolled cell proliferation
		Alterations lead to disrupted cell cycle checkpoints
		Associated with increased risk of metastasis
		Potential therapeutic target for cell cycle intervention
*CDKN2A*	~20–30%	Encodes p16 protein, which inhibits cyclin-dependent kinases
		Deletions or mutations can lead to uncontrolled cell proliferation
		Serves as a potential prognostic marker
		Contributes to understanding tumor-progression mechanisms
*MYC*	~10–20%	Regulates cell proliferation, apoptosis, and metabolic processes
		Overexpression associated with more aggressive tumor behavior
		Potential therapeutic target for molecular interventions
*RUNX2*	~40–50%	Plays a critical role in OS cell differentiation
		Can promote tumor progression and metastasis
		Potential biomarker for understanding tumor development
*HER2*/*ERBB2*	~10–20%	Associated with increased tumor proliferation
		Potential target for targeted therapies
		Correlates with metastatic potential
Homologous Recombination Deficiency (HRD):	~10–15%	OS often demonstrate genomic instability, which can be partially attributed to defects in DNA repair mechanisms
		Some cases of OS exhibit alterations in DNA repair genes, including BRCA1, BRCA2, and other homologous recombination-associated genes
Microsatellite Instability (MSI)	~5%	When present, MSI is often associated with more complex genomic landscapes
		Limited clinical significance has been definitively established in OS
Tumor Mutational Burden (TMB)	TMB in OS can vary widely between individual patients; Estimated TMB ranges typically fall between 5 and 20 mutations per megabase (mut/Mb)	Potential increased responsiveness to immunotherapies
		More complex genetic alterations
		Potentially worse prognosis in some cases
